# Competence-induced protein Ccs4 facilitates pneumococcal invasion into brain tissue and virulence in meningitis

**DOI:** 10.1080/21505594.2018.1526530

**Published:** 2018-09-25

**Authors:** Yujiro Hirose, Masaya Yamaguchi, Kana Goto, Tomoko Sumitomo, Masanobu Nakata, Shigetada Kawabata

**Affiliations:** Department of Oral and Molecular Microbiology, Osaka University Graduate School of Dentistry, Suita, Osaka, Japan

**Keywords:** *Streptococcus pneumoniae*, meningitis, blood-brain barrier, pathogenesis, Ccs4

## Abstract

*Streptococcus pneumoniae* is a major pathogen that causes pneumonia, sepsis, and meningitis. The candidate combox site 4 (*ccs4*) gene has been reported to be a pneumococcal competence-induced gene. Such genes are involved in development of *S. pneumoniae* competence and virulence, though the functions of *ccs4* remain unknown. In the present study, the role of Ccs4 in the pathogenesis of pneumococcal meningitis was examined. We initially constructed a *ccs4* deletion mutant and complement strains, then examined their association with and invasion into human brain microvascular endothelial cells. Wild-type and Ccs4-complemented strains exhibited significantly higher rates of association and invasion as compared to the *ccs4* mutant strain. Deletion of *ccs4* did not change bacterial growth activity or expression of NanA and CbpA, known brain endothelial pneumococcal adhesins. Next, mice were infected either intravenously or intranasally with pneumococcal strains. In the intranasal infection model, survival rates were comparable between wild-type strain-infected and *ccs4* mutant strain-infected mice, while the *ccs4* mutant strain exhibited a lower level of virulence in the intravenous infection model. In addition, at 24 hours after intravenous infection, the bacterial burden in blood was comparable between the wild-type and *ccs4* mutant strain-infected mice, whereas the wild-type strain-infected mice showed a significantly higher bacterial burden in the brain. These results suggest that Ccs4 contributes to pneumococcal invasion of host brain tissues and functions as a virulence factor.

## Introduction

The Gram-positive diplococcus organism *Streptococcus pneumoniae* is a major cause of bacterial pneumonia and meningitis [1,2]. Bacterial meningitis is a significant problem in many areas of the world, especially in developing countries [3], while the mortality rate associated with pneumococcal meningitis has reached 30% in some areas, with sequelae such as hearing loss, focal neurological deficit, and cognitive impairment occurs in approximately 50% of survivors [1,4].

The blood-brain barrier (BBB), formed by a specialized layer of brain microvascular endothelial cells that line cerebral microvessels, impedes influx of most compounds from blood to the brain, thus it regulates macromolecular traffic to maintain biochemical homeostasis in brain tissues. Meningeal pathogens possess an ability to enter the bloodstream and subsequently penetrate the BBB. Pneumococcal penetration of the BBB and invasion into the brain via the bloodstream is also critical for development of meningitis. In *S. pneumoniae*, choline binding protein A (CbpA) [5,6], pneumococcal neuraminidase (NanA) [7–9], and pneumococcal pilus-1 (RrgA) [10,11] each contribute to pneumococcal penetration across the BBB. On the other hand, we reported findings indicating that zinc metalloproteinase ZmpC may have evolved to suppress excess pneumococcal virulence by inhibiting bacterial invasion into central nervous system [12]. Thus, for development of new therapeutic strategies, it is important to elucidate the infectious process of pneumococcal meningitis in greater detail.

*S. pneumoniae* competence is largely controlled by a quorum-sensing system that responds to a self-produced peptide pheromone, designated competence-stimulating peptide (CSP). When CSP is sensed, a transient shift in the transcriptome and proteome pattern is induced, which affects competence as well as pneumococcal virulence [13,14]. This transcriptional shift arises from two overlapping transcription waves of CSP-responsive genes, i.e. “early” and “late” competence genes. The gene encoding candidate combox sites 4 (Ccs4) has been reported to be a late competence gene and *ccs4* deletion had no effect on the transformation efficiency of *S. pneumoniae* [15]. However, previous comprehensive analysis findings revealed that the transcriptional activity of *ccs4* was increased during co-incubation with lung and pharyngeal epithelial cells [16,17]. Additionally, using a mouse model of pneumonia and meningitis, real-time RT-PCR analysis showed increased expression of competence genes following infection of lung and brain tissues [14]. These findings implicate a role of Ccs4 in pneumococcal virulence.

In the present study, we examined the involvement of Ccs4 in *S. pneumoniae* virulence. Deletion of *ccs4* from *S. pneumoniae* attenuated the rates of association with and invasion into human brain microvascular endothelial cells (hBMECs), as well as virulence in a mouse meningitis model. On the other hand, *ccs4* deletion did not change sensitivity to bactericidal activity of whole blood or virulence in a mouse pneumonia model. Our findings suggest the involvement of Ccs4 in development of pneumococcal meningitis.

## Results

### Ccs4 contributes to pneumococcal association to and invasion of hBMECs

**Figure 1.**
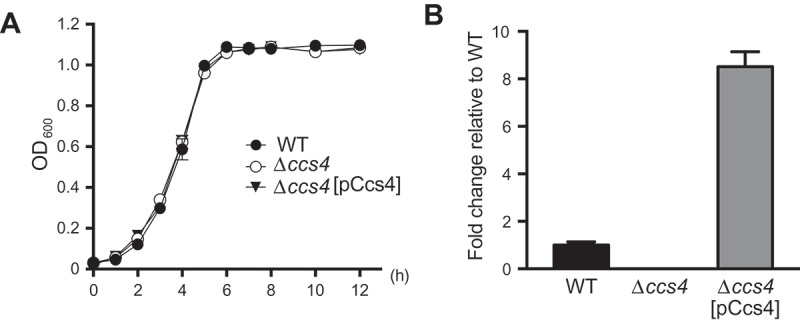
**Effects of *ccs4* deletion on pneumococcal growth and expression of *ccs4***. (a) Growth curves of WT, Δ*ccs4*, and Δ*ccs4* [pCcs4] strains. Values are presented as the mean of 5 samples from a representative experiment. (b) Expression of *ccs4* gene. The level of *ccs4* expression in the Δ*ccs4* and Δ*ccs4*[pCcs4] strains was examined by qPCR and shown relative to that of the WT strain. 16S rRNA was used as the internal control. Values are presented as the mean of 3 samples from the representative of 3 independent experiments. Vertical lines represent the mean + S.E.

**Figure 2.**
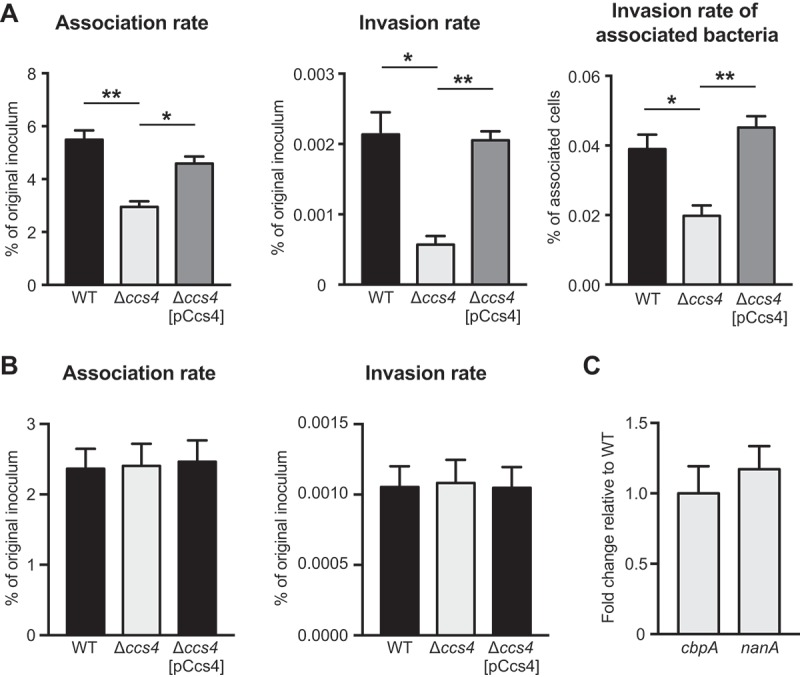
**Pneumococcal interaction with hBMECs and A549 cells**. (a) Rates of association with and invasion into hBMECs by *S. pneumoniae* TIGR4 WT, Δ*ccs4*, and Δ*ccs4*[pCcs4] strains. (b) Rates of association with and invasion into A549 cells by *S. pneumoniae* WT, Δ*ccs4*, and Δ*ccs4*[pCcs4] strains. Association rates were calculated by dividing the CFU value obtained at 1 hour after infection by the value for the original inoculum. Invasion rates were calculated by dividing the CFU value obtained at 1 hour after antibiotic addition by the value for the original inoculum. Values are presented as the mean of 6 wells from one of 3 independent experiments. Vertical lines represent the mean + S.E. Statistical differences between groups were analyzed using a Kruskal-Wallis test with Dunn’s post hoc test. (c) Expression levels of *nanA* and *cbpA*. The *nanA* and *cbpA* gene expression levels in the Δ*ccs4* strain were examined by qPCR, and shown relative to that of the WT strain. 16S rRNA was used as the internal control. Data are presented as the mean of 3 representative samples from the representative of 3 independent experiments. Vertical lines represent the mean + S.E. *p < 0.05 and **p < 0.01.

In this study, we investigated the properties and functions of *S. pneumoniae* Ccs4. First, proteins similar to Ccs4 were searched using the BLASTp program (http://www.ncbi.nlm.nih.gov) with the amino acid sequence of Ccs4 as the search query. Similar proteins were predicted in all *S. pneumoniae* strains for which the complete genome sequences were available. As for other species, limited strains contain proteins similar to Ccs4, including *Streptococcus pseudopneumoniae* (IS7493), *Streptococcus mitis* (B6, KCOM 1350, SVGS_061), and *Streptococcus oralis* (Uo5, S.MIT/ORALIS-351) (Table S1). Therefore, Ccs4 is the molecule specific to mitis group streptococci. Next, to examine the role of *S. pneumoniae* Ccs4 in disease pathogenesis, we constructed a *ccs4* deletion mutant strain (Δ*ccs4*) and its complement strain by transformation with pCcs4 (Δ*ccs4*[pCcs4]). Growth activities of the WT, Δ*ccs4*, and Δ*ccs4*[pCcs4] strains were not significantly different (Figure 1(a)). The *ccs4* gene expression was detected in the WT and Δ*ccs4*[pCcs4] strains, with that in Δ*ccs4*[pCcs4] strain approximately 8-fold greater (OD_600_ = ~ 0.5) (Figure 1(b)). We also performed assays of association and invasion. The Δ*ccs4* strain showed significantly lower rates of association with and invasion into hBMECs as compared to the WT and Δ*ccs4*[pCcs4] strains (Figure 2(a)), while pneumococcal association with and invasion into A549 cells were comparable between the WT and Δ*ccs4* strains (Figure 2(b)).

Some pneumococcal cell surface proteins have been identified to function as adhesins. CbpA binds to the cerebral endothelial laminin receptor and platelet-activating factor receptor [5,6], and those interactions induce pneumococcal invasion into the host brain. NanA, which localizes on bacterial cell surfaces by its cell-wall anchoring motif, activates hBMECs via its lectin-like domain and increases pneumococcal invasion into hBMECs [7,8]. Therefore, it was considered important to assess pneumococcal *cbpA* and *nanA* expressions of the Δ*ccs4* strain. Expression analysis of *cbpA* and *nanA* in exponential phase bacterial cultures was conducted using qPCR. Both genes were found to be comparably expressed in the WT and Δ*ccs4* strains (Figure 2(c)). Thus, deletion of *ccs4* did not alter *cbpA* or *nanA* expression in *S. pneumoniae*.

These results suggest that Ccs4 contributes to pneumococcal association with and invasion into hBMECs, and that Ccs4 may possess hBMEC tropism. The WT and Δ*ccs4*[pCcs4] strains were approximately equal in their ability to associate with and invade hBMECs, while the Δ*ccs4*[pCcs4] strain showed a higher level of *ccs4* expression than the WT strain. There may be an allometric relationship between *ccs4* expression level and pneumococcal virulence, or the level of *ccs4* expression by the WT strain could be adequate for an association with hBMECs.

**Figure 3.**
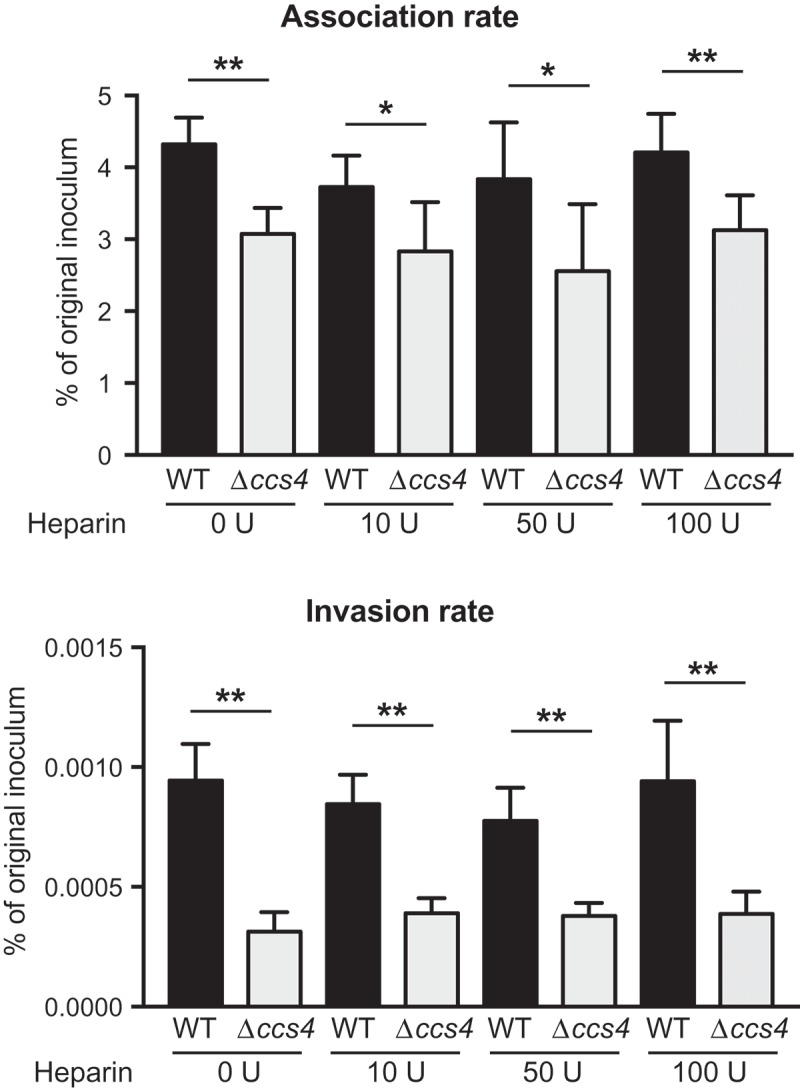
**Effects of heparin pretreatment on association with and invasion into hBMECs by *S. pneumoniae***. Association rates were calculated by dividing the CFU value obtained at 1 hour after infection by the value for the original inoculum. Invasion rates were calculated by dividing the CFU value obtained at 1 hour after antibiotic addition by the value for the original inoculum. Values are presented as the mean of 6 wells from one of 3 independent experiments. Vertical lines represent the mean + S.E. Statistical differences between groups were analyzed using Mann-Whitney’s *U*-test. *p < 0.05 and **p < 0.01.

GNA2132, a surface-exposed protein of *Neisseria meningitidis*, has been reported to possess an arginine-rich region (-**R**F**RR**SA**R**S**RR**S-), which promotes bacterial survival in human serum by binding with heparin [18]. *Streptococcus agalactiae* penetrates the BBB via interactions between bacterial surface alpha C protein and host surface heparan sulfate chains [19]. It has been shown that heparan sulfate and heparin are defined by similar underlying backbones (GlcNAcα1-4GlcAβ1-4/IdoAα1-4)_n_, though heparin undergoes more extensive sulfation and uronic acid epimerization [20]. *S. pneumoniae* Ccs4 also contains an arginine-rich region (-**R**G**R**SA**RR**S**RR**E-). Ccs4 was predicted to be an 8–10-pass transmembrane protein by transmembrane prediction analysis using HMMTOP 2.0, SOSUI engine ver. 1.11, TMHMM Server, v2.0, SMART, and ALOM. The SOSUI algorithm indicated that Ccs4 possesses an extracellular arginine-rich region (Fig. S1). Accordingly, we examined whether pre-incubation of bacteria with heparin had effects on association and invasion rates, though no obvious effect following pre-incubation with heparin was noted (Figure 3). These results suggest that association and invasion are not mediated by interactions between Ccs4 and cell-surface heparan sulfate chains.

### Deletion of *ccs4* gene did not change bacterial survival in mouse blood, susceptibility to LL-37, or biofilm formation ability

**Figure 4.**
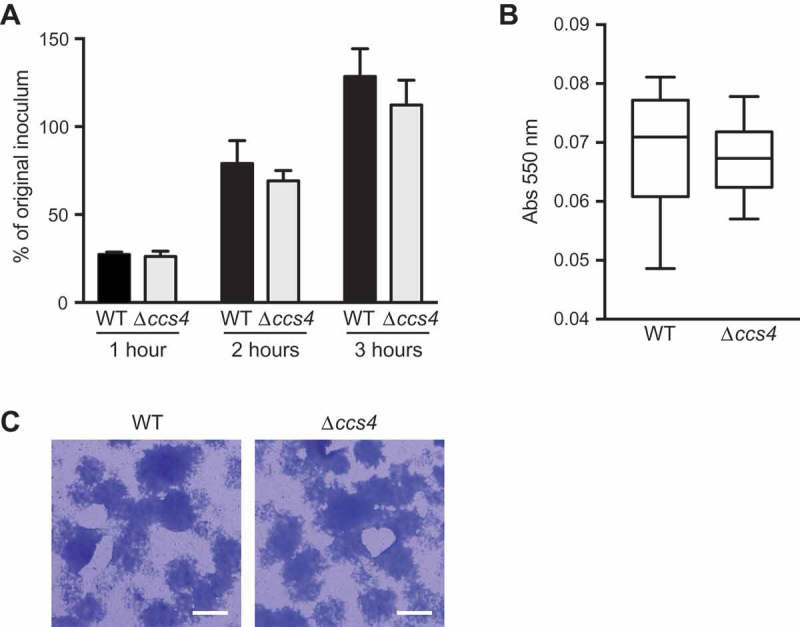
**Bacterial survival in mouse blood and biofilm formation ability**. (a) The *ccs4* gene deletion had no effects on pneumococcal survival in mouse blood. Bacteria were incubated in heparinized mouse blood at 37°C for 1, 2, or 3 hours in a 5% CO_2_ atmosphere. Survival rate was calculated by dividing the CFU value after the period of incubation by the CFU value of the original inoculum. Values are presented as the mean of 6 wells from one of 3 independent experiments. Vertical lines represent the mean + S.E. (b) Effect of *ccs4* deletion on pneumococcal biofilm formation was assessed following incubation in THY at 37°C for 16 hours. Biofilm formed in 96-well microtiter plates was stained with Gram’s stain solution and absorbance was measured at 550 nm. The experiments were performed 3 times and data shown represent the mean of 6 wells from one of 3 independent experiments. Data are displayed as a box-whisker plot (min-[lower quartile–median-upper quartile]-max). Statistical differences between groups were analyzed using Mann-Whitney’s *U*-test. (c) Representative microscopic images of bacterial aggregation from WT strain-derived biofilm (left), and Δ*ccs4* strain mutant-derived biofilm (right). Scale bars indicate 100 μm.

Pneumococcal evasion of innate host defenses, such as bactericidal activities in blood, as well as antimicrobial peptides contribute to development of bacteremia and meningitis. To elucidate whether Ccs4 facilitates escape from innate immunity, bactericidal testing with mouse whole blood was performed (Figure 4(a)). There was no significant difference between the survival rates of the WT and Δ*ccs4* strains in blood after 1, 2, and 3 hours, indicating that pneumococcal Ccs4 does not contribute to its survival in whole blood.

Many bacteria are able to modify their cell surfaces to reduce a negative charge, which is one of their mechanisms of resistance against host cationic antimicrobial peptides [21]. Bioinformatics analysis predicted that Ccs4 possesses a positively charged extracellular region (Fig. S1). Hence, we speculated that the exposed positively charged region of Ccs4 would alter the bacterial surface charge and examined susceptibility to LL-37, a cationic antimicrobial peptide. However, there were no remarkable differences between the WT and *∆ccs4* strains (Table 2). Furthermore, Ccs4 was not shown to be involved in pneumococcal biofilm formation (Figure 4(b,c)). It is possible that the positively charged extracellular region of Ccs4 did not have effects on charge distribution on the cell surface.

**Table 1.** PCR primers used in this study.

Designation	Sequence (5ʹ to 3ʹ)
**For deletional mutagenesis**	
ccs4KOuF	AGCTTATCCCGACCTTCTTTCTG
ccs4KOuR	GTATTCAAATATATCCATCGTTTCTCCTGTTCAATTTATC
ccs4KOdF	ATTATAAAAAAATTGATACTGCGACTCTTTATCGTAAGAG
ccs4KOdR	GGGTGTCACATCCATAACCTTG
ccs4KOaad9F	GAACAGGAGAAACGATGGATATATTTGAATACATACGAAC
ccs4KOaad9R	TAAAGAGTCGCAGTATCAATTTTTTTATAATTTTTTTAATC
**For construction of plasmid used for ectopic expression of Ccs4**	
Ccs4F+ pDCerm	GTTGCATGCGGTACCATGAGTGTGTATGGTAGAGTAGAAG
Ccs4R+ pDCerm	GCGCATGCTAAGCTTTCATCCCATGCATGAGATAGCATCT
pDCermF+ Ccs4	TCATGCATGGGATGAAAGCTTAGCATGCGCTGAAGCGAAG
pDCermR+ Ccs4	ACCATACACACTCATGGTACCGCATGCAACCTCTGTTTGT
pDCermUinivF	GAAGAAAAGAGCTTTGCTAGG
pDCermUinivR	ATCTCCAATCATAAAAATAAC
**Real-time RT-PCR primers**	
TIGR4ccs4F	TCCTTTCTTATTCGCAGCCTTT
TIGR4ccs4R	CCTTATCATGGCTTGGATTGC
TIGR416srRNAF	TGTAGCGGTGAAATGCGTAGATA
TIGR416srRNAR	CAAGCCAGAGAGCCGCTTT
TIGR4nanAF	ATTTGACCCCCAAACCGTATT
TIGR4nanAR	ATGTAACGCATGTGCAAATCG
TIGR4cbpAF	CGACCTCTTTCCTTGCCTTATC
TIGR4cbpAR	GGCAGAACAAGGAGAACAACCT

**Table 2.** MIC and MBC of LL-37 against each strain.

	WT	Δ*ccs4*	Δ*ccs4*[pCcs4]
MIC (μg/ml)	16	16	16
MBC (μg/ml)	32	32	32

### Ccs4 facilitates pneumococcal invasion into brain tissue and virulence

**Figure 5.**
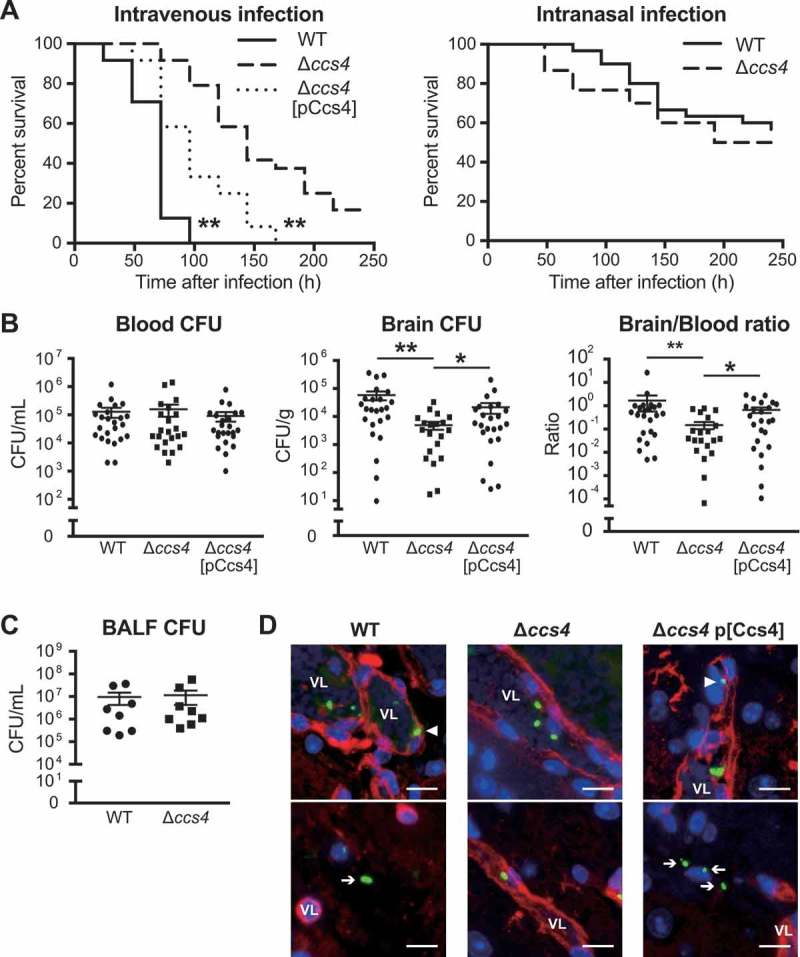
**The *ccs4* gene deletion decreases pneumococcal pathogenesis *in vivo* and invasion into brain**. CD-1 mice were infected with the *S. pneumoniae* TIGR4 WT, Δ*ccs4*, or complement strain. (a) Intravenous injections were performed with ~ 6.0 × 10^6^ CFU in 100 μL of PBS and intranasal injections with ~ 7.5 × 10^7^ CFU in 20 μL of PBS. Mouse survival was monitored for 10 days. Statistical differences between groups were analyzed using a log-rank test. **p < 0.01 versus Δ*ccs4* strain-infected mice. (b) In the intravenous infection model, bacterial burden in the blood and brain were assessed after 24 hours of infection. S.E. values are represented by vertical lines. Statistical differences between groups were analyzed using a Kruskal-Wallis test with Dunn’s post hoc test. *p < 0.05 and **p < 0.01. (c) Bacterial burden in bronchoalveolar lavage fluid (BALF) was assessed at 24 hours after infection in pneumonia model mice. Values are presented as the mean of 8 samples from 2 independent representative experiments. Vertical lines represent the mean ± S.E. Statistical differences between groups were analyzed using Mann-Whitney’s *U*-test. (d) Representative microscopic images of brain vascular tissues from WT (left), *ccs4* mutant (center), and complement (right) strain-infected mice showing staining for *S. pneumoniae* TIGR4 (green), brain vascular endothelial cells (red), and nuclei (blue). Triangles indicate *S. pneumoniae* binding or invasion of brain vascular endothelial cells. Arrows indicate *S. pneumoniae* invading the brain tissue. Vessel lumens are marked with VL. Scale bars indicate 10 μm. *p < 0.05 and **p < 0.01 versus control.

*S. pneumoniae* is known to be the main cause of community-acquired pneumonia and our results noted above suggest that Ccs4 contributes to the process of pneumococcal meningitis development. Hence, we also investigated the role of Ccs4 *in vivo* in mice following intranasal infection as a model of pneumonia and intravenous infection as a model of meningitis infection. In mice that received intranasal infection, there were no statistical differences in regard to survival time or bacterial burden in bronchoalveolar lavage fluid between those infected with the WT and Δ*ccs4* strain (Figure 5(a,c)). On the other hand, intravenous infection model mice infected with the Δ*ccs4* strain showed significantly lower levels of virulence as compared to mice infected with the WT and Δ*ccs4*[pCcs4] strains. All mice infected with WT and Δ*ccs4*[pCcs4] strains died within 168 hours, whereas some of the Δ*ccs4* strain-infected mice survived for at least 240 hours (Figure 5(a)). In addition, we examined bacterial burden in brain and blood samples obtained at 24 hours after intravenous infection (Figure 5(b)). WT and Δ*ccs4*[pCcs4] strain-infected mice showed a significantly higher bacterial burden in the brain as compared to mice infected with the Δ*ccs4* strain, whereas that in blood was comparable among the 3 groups. The median ratio of brain/blood CFU for the WT and Δ*ccs4*[pCcs4] strain-infected mice was also significantly greater than that of those infected with the Δ*ccs4* strain. Therefore, using the present intravenous infection model, we conducted immunofluorescence staining of *S. pneumoniae* and brain vascular endothelial cells in brain tissues to investigate whether the ∆*ccs4* strain binds to the BBB at a lower level of efficiency as compared to the WT and Δ*ccs4*[pCcs4] strains (Figure 5(d)). Immunofluorescence imaging showed that both WT and Δ*ccs4*[pCcs4] had greater levels of binding to brain vascular endothelial cells and invasion into brain tissues as compared to the ∆*ccs4* strain. These observations were consistent with the present quantitative bacterial burden results (Figure 5(b,d)).

In the present intravenous infection model, the Δ*ccs4*[pCcs4] strain showed significantly higher levels of virulence and bacterial burden in excised brains as compared to the Δ*ccs4* strain, whereas its pathogenicity was lower as compared to the WT strain (Figure 5(a,b)). Hence, blood and brain homogenates isolated from Δ*ccs4*[pCcs4] strain-infected mice were seeded onto THY agar with or without erythromycin to estimate the percentage of Δ*ccs4* strains with a Ccs4-expressing vector. Erythromycin-resistant *S. pneumoniae* bacterial cells containing a Ccs4-expressing vector were found to be slightly decreased in blood and brain tissues (Fig. S2). Loss of plasmid-carrying strains may result in partial recovery of pathogenicity of the Δ*ccs4*[pCcs4] strain *in vivo*.

**Figure 6.**
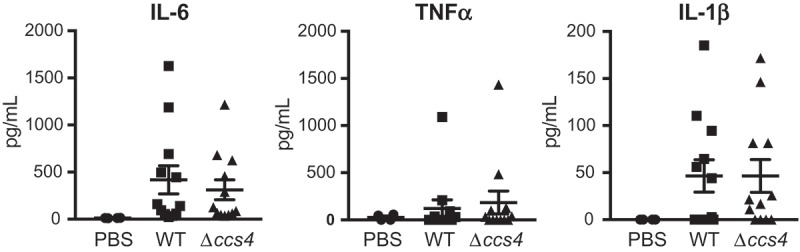
**Levels of proinflammatory cytokines in mouse plasma**. Plasma samples were collected from intravenous infection model mice at 24 hours after infection. Values are presented as the mean of 12 samples. Vertical lines represent the mean ± S.E. Statistical differences between groups were analyzed using Mann-Whitney’s *U*-test.

Stimulation of brain endothelial cells with proinflammatory cytokines leads to a decrease in barrier integrity [22]. Hence, we quantified the levels of IL-6, TNF-α, and, IL-1β in plasma obtained from intravenous infection model mice. However, there were no significant differences between the levels of cytokines in plasma between the WT strain- and Δ*ccs4* strain-infected mice (Figure 6). These results indicate that Ccs4 functions as a virulence factor by contributing to pneumococcal invasion into brain tissue and development of pneumococcal meningitis, in a manner independent of proinflammatory cytokines.

## Discussion

CSP controls the natural genetic transformation of *S. pneumoniae* [13], with two overlapping waves of transcription of CSP-responsive genes, “early” and “late” competence genes. Those early genes participate in competence regulation and link signals to late gene expression [23], while deletion of late genes, such as *coiA, celA*, and *celB*, abolishes transformation [15,24]. Although it has been reported that deletion of late genes, including *lytA, cibAB*, and *cbpD*, decreased pneumococcal virulence in mouse models of pneumonia and bacteremia, many of the genes in that study were hypothetical or conserved hypothetical proteins, and their roles in pneumococcal pathogenesis are unknown [25]. Previous comprehensive analysis findings showed that *S. pneumoniae ccs4* expression is upregulated when the bacteria make contact with host cells, including pharyngeal epithelial [17] and alveolar epithelial [16] cells. Therefore, there is a possibility that Css4 is expressed by bacteria in contact with the BBB. The present study is the first to focus on the function of Ccs4 in disease pathogenesis. Ccs4 was found to have no effects on pneumococcal survival in blood or establishment of pneumonia, nevertheless it contributes to pneumococcal invasion of brain tissue and development of meningitis.

The BBB is composed of endothelial cells lining cerebral microvessels. Since meningitis develops following bacterial adhesion to and invasion through the BBB, researchers have used hBMECs to study the pathogenesis of bacterial meningitis [22,26]. Bacterial meningitis pathogens are able to penetrate the BBB transcellularly, paracellularly and/or in infected phagocytes [27]. Transcellular traversal of the BBB has been demonstrated for *S. pneumoniae* [22]. However, though pneumococcal paracellular penetration of the BBB has been suggested [22,28]. those findings have not been verified. In the present study, we found a significantly lower rate of hBMEC invasion by the Δ*ccs4* strain, suggesting that Ccs4 facilitates transcellular penetration by *S. pneumoniae*.

Several studies have reported bacterial virulence factors that contribute to association with and invasion into hBMECs, including human pathogens such as *Streptococcus agalactiae* (Lmb, IagA, LTA, FbsA, SfbA, PilA, PilB, Srr-1, HvgA, and ACP), *Escherichia coli* (FimH, Nlp1, OmpA, CNF-1, IbeA, Ibe10, AslA, Tra, YijP, and Flagella), and *S. pneumoniae* (CbpA/PspC, phosphorylcholine, NanA, and RrgA) [10,11,22]. It has been reported that polymeric immunoglobulin receptor and platelet endothelial cell adhesion molecules bind to the pneumococcal adhesins RrgA and CbpA/PspC, resulting in mediation of bacterial brain invasion [11]. No homologous sequence of those molecules was detected in Ccs4. Despite abundant epidemiological data that *S. pneumoniae* is the most common cause of bacterial meningitis [3], only four pneumococcal factors have been reported to contribute to BBB penetration. Furthermore, NanA, CbpA, and RrgA are not considered to be competence-inducible molecules [25]. Therefore, the present results indicate that pneumococcal Ccs4 is a novel factor in regard to association with and invasion into hBMECs, and are the first to show a relationship of competence-induced molecules with that association and invasion against hBMECs.

Cell adhesion molecules, extracellular matrices, molecular receptors, and glycoproteins are known to be host receptors that interact with bacterial virulence factors, thus contributing to association with and invasion into hBMECs [22]. Heparan sulfate proteoglycan, one of those glycoproteins, has been found on cell surfaces and in the extracellular matrix [29,30]. The glycosaminoglycans heparin and heparan sulfate contain similar structural units. Binding of heparin via an arginine-rich region is a characteristic of *Neisseria meningitidis* strain GNA2132 [18]. Although Ccs4 also possesses an arginine-rich region, our results suggest that it does not bind heparin. Excluding the arginine-rich region, no other similar sequences were found in a comparison between GNA2132 and Ccs4. We consider that another mechanism of host-bacterial interaction, excluding heparan sulfate-Ccs4 interaction, contributes to hBMEC association and invasion.

Infection with the Gram-negative bacterium *E. coli* is also an important cause of bacterial meningitis [3], and the transmembrane proteins, such as OmpA [31] and YijP [32], contribute to hBMEC adhesion and invasion. OmpA loops, which mediate bacterial entry into hBMECs, have been identified [31,33]. Thus, it is possible that a transmembrane protein facilitates the association with and invasion into hBMECs. The Gram-positive bacterium *S. pneumoniae* has both a cell wall and capsule surrounding its membrane. Despite prediction that Ccs4 is a transmembrane protein, we found that Ccs4 facilitated interaction between *S. pneumoniae* and hBMECs, thus it is important whether there are possible opportunities for exposure of Ccs4 on the bacterial surface. A defect of capsular polysaccharide on pneumococcal surfaces during adherence to and invasion into epithelial cells has been reported [34]. In addition, shedding of the capsule is dependent on the pneumococcal autolysin LytA, which is induced by LL-37 stimulation [35]. LytA is dispersed circumferentially around the cell and induces release of peptidoglycan sorted proteins including CbpA and pilus. LytA, an *N*-acetylmuramyl-l-alanine amidase, is important for peptidoglycan remodeling during growth [36] and appears to be constitutively expressed [37,38]. Death or suicide of *S. pneumoniae* in the stationary phase has been attributed to autolysis by LytA [38,39], however, it has been proposed that spontaneous pneumococcal death is due to hydrogen peroxide (H_2_O_2_), a by-product of aerobic metabolism by SpxB, not LytA [40]. Taken together, not only cell wall attached proteins but also exposed Ccs4 may interact with host cells when host-pneumococcal contact induces LytA expression, causing degradation of the cell wall peptidoglycan and capsule. Interestingly, *lytA* has also been classified as a late competence gene [25]. Furthermore, Ccs4 may interact with host cells following expression of LytA induced by host-pneumococcal contact.

Our results suggest that Ccs4 possesses tropism for brain endothelial cells. Endothelial cells express cell-type-specific tyrosine kinase receptors, including vascular endothelial growth factor, Tie, and Eph receptors [41,42], while alveolar epithelial cells also express specific molecules [43]. A549 cells are type 2 alveolar cells and synthesize surfactant proteins [44]. Thus, there are varieties of types of molecules on the surfaces of A549 cells and hBMECs, which may have effects on Ccs4 tropism both *in vivo* and *in vitro*.

Ccs4 has been reported to be upregulated during the late competence stage [45]. Allolysis caused by late competence genes encoding CbpD, LytA, and CibAB is an important virulence trait of *S. pneumoniae* [46]. ComX, a σ factor, up-regulates the *ccs4* gene in addition to allolysis genes, thus it is possible that Ccs4 is involved in competence-dependent allolysis. However, the molecule that interacts with Ccs4 remains unclear and the exact roles of Ccs4 require further analysis.

We investigated protein motifs in the Ccs4 sequence using a MOTIF search (http://www.genome.jp/tools/motif/), though no known motifs were revealed. Additionally, a protein similar to Ccs4 is predicted only in *S. pneumoniae* and genetically closely related species (Table S1)[47]. Our results suggest that pneumococcal Ccs4 has an unknown motif and may be a virulence factor peculiar to mitis group streptococci.

In conclusion, this is the first report of the function of Ccs4 in invasion into host brain tissue. A better understanding of the mechanism of pneumococcal brain invasion will allow more detailed discussion regarding challenges and opportunities for development of therapeutic strategies.

## Materials and methods

### Bacterial strains and culture conditions

*Streptococcus pneumoniae* TIGR4 (accession: AE005672.3) wild-type strain (WT) and its derivative strains were cultured in Todd-Hewitt broth (BD Biosciences) supplemented with 0.2% yeast extract (BD Biosciences) (THY) with or without antibiotics at 37°C. For growth measurements, overnight cultures of each strain were back-diluted 1:50 into fresh THY and grown at 37°C. Growth was monitored by measuring optical density at 600 nm (OD_600_) every 0.5–1 hour.

*Escherichia coli* strain XL10-Gold (Agilent Technologies) was used as a host for derivatives of the pDCerm plasmid containing the erythromycin-resistance cassette [48]. *E. coli* strains were cultured in Luria-Bertani broth (LB) (Nacalai) at 37°C with agitation. For selection and maintenance of recombinant strains, antibiotics were added to the medium at the following concentrations: erythromycin (Sigma-Aldrich), 400 μg/mL for *E. coli* and 5 μg/mL for *S. pneumoniae*; spectinomycin (Wako), 120 μg/mL for *S. pneumoniae*.

### Construction of *ccs4* mutant and complement strains

An *S. pneumoniae ccs4* (locus_tag: SP_0200, GenBank: AAK74380.1) mutant strain (Δ*ccs4*) was constructed as previously described [49,50]. Briefly, the upstream region of *ccs4*, an *aad9* cassette, and the downstream region of *ccs4* were combined by PCR, then the combined product was used to obtain mutant strains. An *S. pneumoniae* TIGR4 isogenic mutant strain was constructed using a double crossover recombination technique with CSP [51].

A Ccs4-expressing vector (pCcs4) was constructed by assembly of the *ccs4* gene and pDCerm using GeneArt® Seamless Cloning and Assembly Enzyme Mix (Thermo Fisher Scientific). The pCcs4 was introduced into the *S. pneumoniae* TIGR4 Δ*ccs4* strain using CSP as described above. The primers used are shown in Table 1.

### S. pneumoniae *association and invasion assays*

Bacterial association with and invasion into hBMECs or the human alveolar cell line A549 were quantified as previously described, with minor modifications [12,19,26,52]. hBMECs or A549 cells were seeded at 2 × 10^5^ cells into 24-well plates 1 day prior to bacterial infection. In each well, ~ 2.0 × 10^6^ CFU of bacteria from exponential phase bacterial cultures (OD_600_ = ~ 0.5) was added to infect hBMECs, while bacteria at ~ 1.0 × 10^7^ CFU was added to infect A549 cells. For assays using heparin, *S. pneumoniae* organisms were pre-incubated with 10, 50, or 100 U of heparin or buffer for 30 minutes prior to infection. To determine bacterial association, hBMECs and A549 cells were infected with the bacteria for 1 and 2 hours, respectively, then the cells were washed and harvested with PBS containing 0.05% trypsin and 0.025% Triton X-100. To examine bacterial invasion, hBMECs and A549 cells were washed following incubation for 1 and 2 hours, respectively, then medium containing 100 μg/mL of gentamicin (Nacalai) was added, after which the both types of cells were incubated for an additional 1 hour and harvested. The number of bacteria in each sample was quantified by serial dilution plating.

### Quantitative real-time PCR

Quantitative real-time PCR (qPCR) was performed as previously described, with minor modifications [12,53]. Bacterial mRNA was extracted from exponential phase bacterial cultures (OD_600_ = ~ 0.5) and 16S rRNA was used as a normalizing control.

### Blood bactericidal assay

A blood bactericidal assay was performed as previously described, with minor modifications [54,55]. Briefly, heparinized mouse blood (190 μL) and exponential phase bacteria (~ 1.5 × 10^6^ CFU in 10 μL of PBS) were mixed in 96-well plates and incubated at 37°C in 5% CO_2_ for 1, 2, or 3 hours. Viable cell counts were determined by plating diluted samples on THY blood agar.

### Biofilm formation assay

Bioﬁlm formation was investigated as previously described, with minor modifications [56]. Overnight bacterial cultures (20 μL) were mixed with 180 μL of THY in 96-well (flat-bottom) microtiter plates (Thermo Fisher Scientific). After the plates were incubated at 37°C for 16 hours, liquid medium was removed and the wells were rinsed with 200 μL of PBS. The plates were then stained with 100 μL of Gram’s stain solution (I) (Sigma-Aldrich) for 1 minute and rinsed with 200 μL of distilled water. Next, the crystal violet was recovered with in 200 μL of 99.5% ethanol for 1 minute and dried. Quantification of stained biofilm was performed by measuring absorbance at 550 nm. Images of the biofilms were captured using microscope prior to recovering.

### Determination of minimum inhibitory concentration (MIC) and minimum bactericidal concentration (MBC) against LL-37

MIC and MBC values were determined as previously described [57,58]. Briefly, 1.0 × 10^4^ bacteria were added to Todd-Hewitt broth supplemented with two-fold serial dilutions of the antimicrobial peptide LL-37 (AnaSpec). Pneumococcal growth after 16 hours at 37°C in an anaerobic condition was spectrophotometrically measured at OD_600_. We defined OD_600_ values less than 0.06 as complete inhibition of bacterial growth. Next, 5 μL samples from each well were inoculated to THY blood agar plates to determine MBC. The concentration of LL-37 at which no growth was detectable was defined as the MBC.

### Mouse model of meningitis and pneumonia

Mouse infection assays were conducted as previously described, with minor modifications [12,53]. All mouse experiments were conducted in accordance with animal protocols approved by the Animal Care and Use Committee of Osaka University Graduate School of Dentistry (24–025-3, 28–002-0). CD-1 (Slc: ICR) mice (6 weeks old, female; SLC) were intravenously infected with ~ 7.5 × 10^6^ CFU in 100 μL of PBS or intranasally with ~ 6.0 × 10^7^ CFU in 20 μL of PBS. Mouse survival was monitored for 10 days.

To assess bacterial burden, mice were euthanized at 24 hours after infection by a lethal intraperitoneal injection of sodium pentobarbital, then blood aliquots and brain samples were collected. We assessed bacterial burden at 24 hours, because the appropriate time point in our previous study to assess BBB binding ability was found to be 24 hours after infection [12]. Bacterial counts in blood and brain homogenates were determined after plating serial dilutions, with those in the brain corrected for differences in brain weight. Blood and brain homogenates isolated from complement strain-infected mice were seeded onto THY agar with or without erythromycin to estimate the percentage of Δ*ccs4* with a Ccs4-expressing vector. To measure cytokines in plasma, mice were euthanized by lethal intraperitoneal injection of sodium pentobarbital and blood aliquots were collected at 24 hours after intravenous infection. Heparinized blood was centrifuged at 2000 × g for 30 minutes and plasma was collected. The concentrations of IL-6, TNF-α, and, IL-1β in plasma were measured by ELISA (R&D Systems), according to the manufacturer’s guidelines. Data obtained from 2 or 3 independent experiments (n = 24) were pooled.

### Bronchoalveolar lavage fluid (BALF) collection

Using a mouse pneumonia model, mice were euthanized by lethal intraperitoneal injection of sodium pentobarbital at 24 hours after infection and BALF was collected by washing the lungs with 1 mL of cold sterile PBS. Viable cell counts were determined by plating diluted samples on THY blood agar.

### Immunofluorescence staining

Brain tissues were fixed in 4% paraformaldehyde at 4°C overnight and embedded in paraffin wax, then 7-μm-thick sections were prepared and subjected to immunofluorescence staining to detect *S. pneumoniae* and brain vascular endothelial cells. Following deparaffinization, sections in a 10-mM sodium citrate solution (pH 6.0) were heated for 5 min in a pressure cooker to retrieve the antigens. To visualize brain endothelial vascular cells, sections were incubated with Dylight 594-conjugated Lycopersicon esculentum lectin (1:100, Vector Labs) for 30 min. To stain *S. pneumoniae* organisms, sections were pre-incubated in blocking solution (PBS containing 2% normal donkey serum) for 30 min at room temperature, then rabbit antisera for *S. pneumoniae* serotype 4 (1:100, Denka Seiken) was applied overnight at 4°C. The next day, an Alexa Fluor® 488-conjugated donkey anti-rabbit polyclonal antibody (1:200, Abcam) was applied for 30 min at room temperature as the secondary antibody. Finally, the sections were counterstained with ProLong Gold Antifade Mountant with DAPI (Life Technologies).

### Statistical analysis

Statistical analysis was performed using GraphPad Prism version 7.0 (GraphPad Software Inc.). Differences between groups were analyzed using a Mann-Whitney *U* test. A Kruskal-Wallis test with Dunn’s post hoc test was used for multiple comparisons. Mouse survival was analyzed with a log-rank test.
