# Supplementary material for: Competence-induced protein Ccs4 facilitates pneumococcal invasion into brain tissue and virulence in meningitis
Source: Virulence. 2018 Sep 25;9(1):1576–87. doi: 10.1080/21505594.2018.1526530 (PMC6177246; doi:10.1080/21505594.2018.1526530)
Supplement: Supplemental Material [file kvir-09-01-1526530-s001.pdf]

Supplementary Information

Competence-induced protein Ccs4 facilitates pneumococcal invasion into brain tissue and virulence in meningitis

Yujiro Hirose[a], Masaya Yamaguchi[a,*], Kana Goto[a], Tomoko Sumitomo[a], Masanobu Nakata[a], Shigetada Kawabata[a]

[a]Department of Oral and Molecular Microbiology, Osaka University Graduate School of Dentistry, Suita, Osaka 565-0871, Japan

*Address correspondence to: Masaya Yamaguchi, yamaguchi@dent.osaka-u.ac.jp

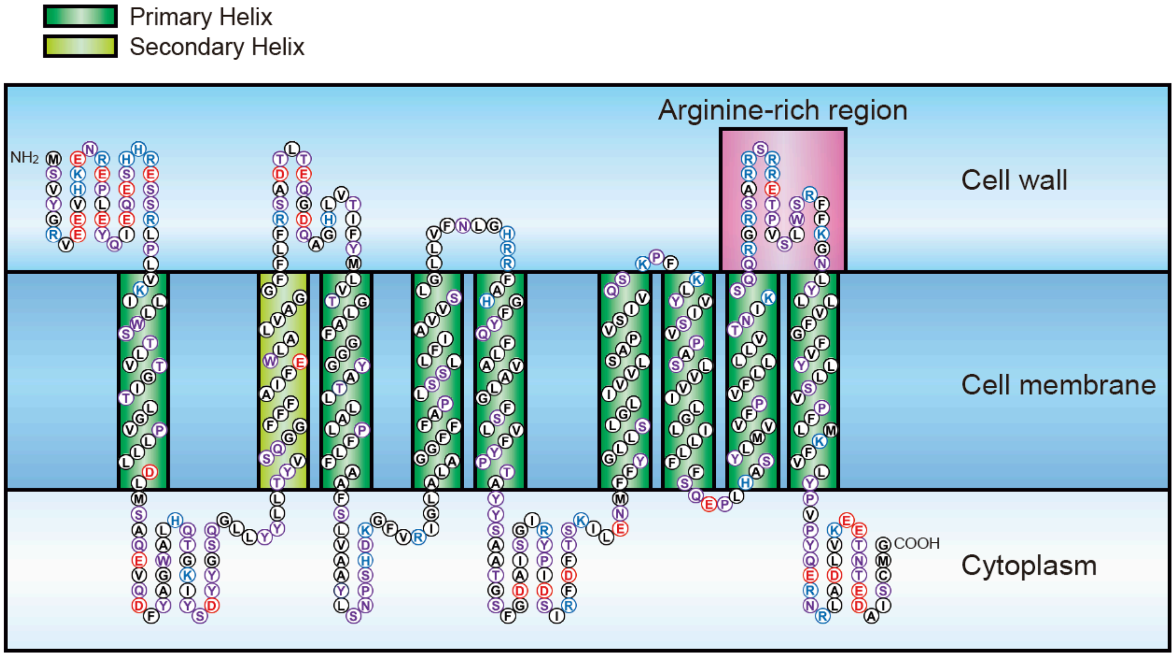

Fgure S1. Deduced transmembrane topology of Ccs4. Transmembrane regions of Ccs4 were predicted using the SOSUI algorithm. Pink-shaded area indicates extracellular region with an arginine-rich region (-**R**F**RR**SA**R**S**RR**S-).

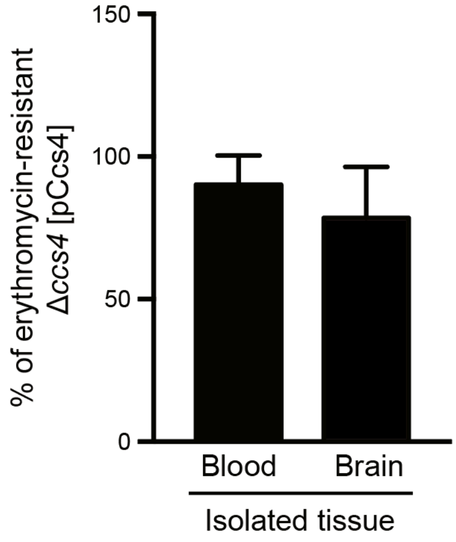

Figure S2. The percentage of Δ*ccs4* containing Ccs4-expressing vector in blood and brain homogenates. In the intravenous infection model, we counted CFU in blood and brain homogenates which isolated from complement strain-infected mice on THY agar with or without erythromycin (n = 18). The percentage of erythromycin-resistant Δ*ccs4* [pCcs4] = (CFU counts on THY ager with erythromycin / CFU counts on THY ager without erythromycin) ×100.

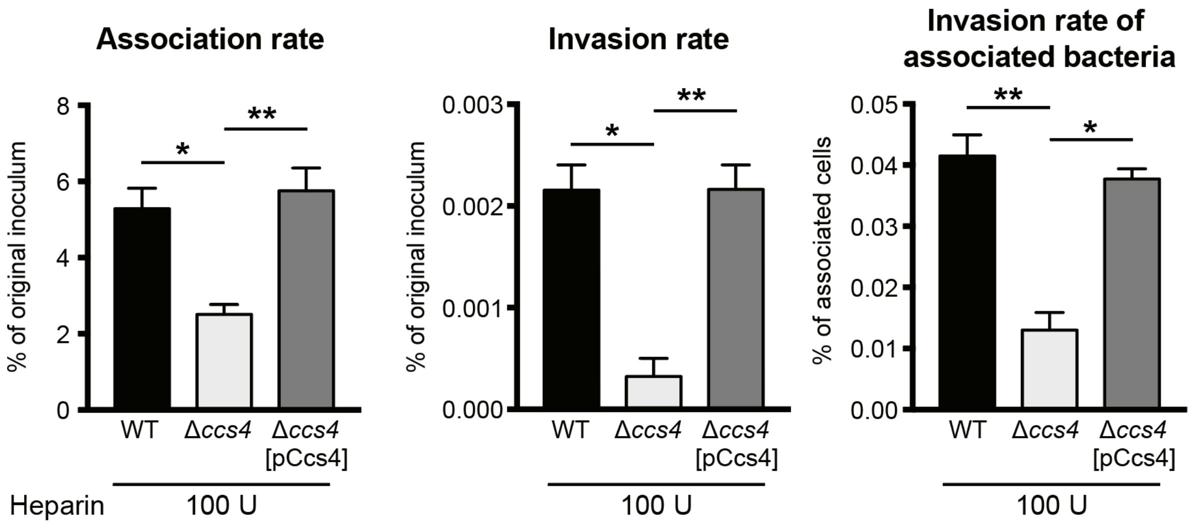

Figure S3. Effects of heparin pretreatment on association with and invasion into hBMECs by *S. pneumoniae*. Association rates were calculated by dividing the CFU value obtained at 1 hour after infection by the value for the original inoculum. Invasion rates were calculated by dividing the CFU value obtained at 1 hour after antibiotic addition by the value for the original inoculum. Values are presented as the mean of 6 wells from one of 3 independent experiments. Vertical lines represent the mean +S.E. Statistical differences between groups were analyzed using a Kruskal-Wallis test with Dunn's post hoc test. *$p < 0.05$ and **$p < 0.01$.

Materials and Methods

Similarity search

We searched for proteins similar to Ccs4 of *S. pneumoniae* TIGR4 using the BLASTP program (NCBI BLAST). Proteins with an E-value <2E-100, coverage of the query >99%, and identity >40% are listed. Evaluation criteria for capsule +/- were used as presented in literature or based on the existence of *cpsA–cpsD* encoding a putative polysaccharide capsule in *S. pneumoniae* TIGR4.

Supplementary Table 1. List of proteins similar to *S. pneumoniae* TIGR4 Ccs4

Bacteria	Strain	Accession number	Protein ID	Product	Query cover	E value	Identity	Capsule	Serotype
S. pneumoniae	TIGR4	AE005672.3	AAK74380.1	Competence-induced protein Ccs4	100%	0	100%	+	4
	AP200	CP002121.1	ADM83843.1	Hypothetical protein	99%	0	100%	+	11A, ST62
	INV200	FQ312029.1	CBW33819.1	Putative membrane protein	99%	0	100%	+	14
	OXC141	FQ312027.1	CBW31886.1	Putative membrane protein	99%	0	100%	+	3
	INV104	FQ312030.1	CBW35848.1	Putative membrane protein	99%	0	100%	+	1
	SPN034183	FQ312043.1	CCP31941.1	Putative membrane protein	99%	0	100%	+	3
	SPN994038	FQ312041.2	CCP29955.1	Putative membrane protein	99%	0	100%	+	3
	SPN994039	FQ312044.2	CCP33914.1	Competence-induced protein Ccs4	99%	0	100%	+	3
	A66	LN847353.1	CRI60910.1	Competence-induced protein Ccs4	99%	0	100%	+	3
	NCTC7465	LN831051.1	COT02612.1	Competence-induced protein Ccs4	99%	0	100%	+	1
	D39	CP000410.1	ABJ53888.1	Competence-induced protein Ccs4	99%	0	99%	+	2
	R6	AE007317.1	AAK98986.1	Hypothetical protein	99%	0	99%	−	−
	CGSP14	CP001033.1	ACB89463.1	Competence-induced protein Ccs4	99%	0	99%	+	14
	Hungary19A-6	CP000936.1	ACA36557.1	Competence-induced protein Ccs4	99%	0	99%	+	19A
	ST556	CP003357.1	AFC93944.2	Damage-inducible protein CinA	99%	0	99%	+	19F
	Taiwan19F-14	CP000921.1	ACO24255.1	Competence-induced protein Ccs4	99%	0	99%	+	19F
	SPNA45	CP000936.1	CCM09114.1	Putative membrane protein	99%	0	99%	+	3
	G54	CP001015.1	ACF56587.1	Hypothetical protein	99%	0	99%	+	19F
	gamPNI0373	CP001845.1	AFS42337.1	Competence-induced protein Ccs4	99%	0	99%	+	1
	TCH8431/19A	CP001993.1	ADI68742.1	Competence-induced protein Ccs4	99%	0	99%	+	19A
	ATCC700669	FM211187.1	CAR68052.1	Putative membrane protein	99%	0	99%	+	23F
	P1031	CP000920.1	ACO20493.1	Competence-induced protein Ccs4	99%	0	99%	+	1
	70585	CP000918.1	ACO17022.1	Competence-induced protein Ccs4	99%	0	99%	+	5
	670-6B	CP002176.1	ADM90353.1	Competence-induced protein Ccs4	99%	0	99%	+	6B
	NT_110_58	CP007593.1	AJD71143.1	Hypothetical protein	99%	0	99%	−	−
	JJA	CP000919.1	ACO18425.1	Competence-induced protein Ccs4	99%	0	99%	+	19F
	SPN034156	FQ312045.1	CCP36950.1	Putative membrane protein	99%	0	99%	+	3
S. pseudopneumoniae	IS7493	CP002925.1	AEL09792.1	Competence-induced protein Ccs4	99%	0	96%	−	−
S. mitis	B6	FN568063.1	CBJ23149.1	Competence-induced protein Ccs4	99%	0	91%	−	−
	KCOM 1350	CP012646.1	ALD67418.1	Damage-inducible protein CinA	99%	6.00E-133	54%	−	−
	SVGS_061	CP014326.1	AMH89424.1	Damage-inducible protein CinA	99%	4.00E-124	53%	+	−
S. oralis	Uo5	FR720602.1	CBZ01433.1	Competence-induced protein Ccs4	99%	1.00E-108	48%	+	−
	S.MIT/ORALIS-351	CP019562.1	AQA08377.1	Putative membrane protein	99%	1.00E-108	49%	+	−
S. parasanguis	DORA_23_24	AZMG01000719.1	ETJ06019.1	Competence-induced protein Ccs4	99%	1.00E-132	48%	−	−

(E-value < 2E-100, Coverage of the query > 99%, Identity > 48%)
